# Task-Parametrized Dynamics: Representation of Time and Decisions in Recurrent Neural Networks

**DOI:** 10.1101/2025.09.15.676356

**Published:** 2025-09-19

**Authors:** Cecilia Jarne, Ryeongkyung Yoon, Tahra Eissa, Zachary P. Kilpatrick, Kresimir Josić

**Affiliations:** 1 Universidad Nacional de Quilmes, Argentina.; 2 CONICET, Buenos Aires, Argentina.; 3 Center of Functionally Integrative Neuroscience, Department of Clinical Medicine, Aarhus University, Aarhus, Denmark.; 4 Latin American Brain Health Institute (BrainLat), Universidad Adolfo Ibàñez (UAI), Santiago, Chile.; 5 Department of Applied Mathematics, University of Colorado Boulder, Boulder, Colorado, 80309, USA.; 6 Department of Physiology and Biophysics, University of Colorado School of Medicine, Aurora, Colorado, 80045, USA.; 7 Department of Mathematics and Computer Science, Wabash College, Crawfordsville, IN, USA.; 8 Department of Mathematics, University of Houston, Houston, TX, 77204, USA.; 9 Department of Biology and Biochemistry, University of Houston, Houston, TX, 77204, USA.

**Keywords:** RNNs, Temporal Representation, Decision Making, Solution Degeneracy

## Abstract

How do recurrent neural networks (RNNs) internally represent elapsed time to initiate responses after learned delays? To address this question, we trained RNNs on delayed decision-making tasks of increasing complexity: binary decisions, context-dependent decisions, and perceptual integration. We analyzed RNN dynamics after training using eigenvalue spectra, connectivity structure, and population trajectories, and found that 1) distinct dynamical regimes emerge across networks trained on the same task whereby oscillatory dynamics support precise timing, and integration supports evidence accumulation, 2) a population-wide representation of time and decision variables emerges rather than dedicated sub-populations to tracking time and other task-specific variables; and 3) the neural trajectories align only with the output weights near decision points, as shown by trajectory readout correlations, revealing task-driven coordination of precisely timed task representation and readout. These results show that RNNs can use either integration or oscillations to represent time, and highlight how structured connectivity enables diverse solutions to temporal computation problems, consistent with biological principles of degeneracy and functional redundancy.

## 1 Introduction

Timing is critical for humans and animals to navigate their environment and make the rapid choices necessary for survival (Paton and Buonomano 2018; Jazayeri and Shadlen 2010). Recurrent Neural Networks (RNNs) have become a central modelling framework in computational neuroscience, and have been used to uncover potential mechanisms that biological networks use to process temporal and sensory information and generate decisions (Mante et al. 2013; Russo et al. 2020; Laje and Buonomano 2013; Buonomano and Maass 2009). The ability of RNNs to generate rich, high-dimensional dynamics makes them particularly well-suited for understanding the range of mechanisms that neural networks can use to integrate multiple time-varying inputs and for uncovering latent neural computations (Barak 2017; Maheswaranathan et al. 2019; Mastrogiuseppe and Ostojic 2018). Much previous work has been devoted to understanding how context and decision variables are represented in RNN population activity (Mante et al. 2013; Remington et al. 2018). Despite the possibility of high-dimensional RNN dynamics, many studies have shown that, after training, network activity converges to a small set of dominant trajectories or low-dimensional manifold (Vyas et al. 2020). Yet, there is still no consensus on how networks concurrently represent and update multiple dynamically evolving, task-dependent variables. In particular, how RNNs can simultaneously encode elapsed time and decision variables during delayed decision-making tasks, and what underlying dynamical mechanisms allow them to do so, remains unclear.

How time is represented in RNNs and biological networks is not fully understood (Merchant et al. 2013a), since the encoding of time depends on both the specific cognitive process being modelled and how the task is parameterized. For instance, in RNNs trained to generate variable time intervals, temporal scaling (i.e., temporal compression/expansion of neural trajectories while preserving their shape) can depend on the strength of external input (Hardy et al. 2018). These findings are consistent with experiments where non-human primates trained to report time intervals show heterogeneous temporal dynamics within cortical populations but unified population trajectories that show comparable temporal scaling (Wang et al. 2018). This same population-level temporal scaling has also been observed in other brain regions, including striatal neurons (Mello et al. 2015), supplementary motor area (Merchant et al. 2013b) and hippocampal time cells (MacDonald et al. 2011; Wang et al. 2018; Merchant et al. 2013b). Beyond hippocampal time cells, (Harvey et al. 2012) exemplify sequence generation as a timing mechanism in cortex. Likewise, both RNNs and biological neural circuits can perform timing and decision-making tasks by engaging diverse computational mechanisms and neural population dynamics (Vyas et al. 2020), including ramping activity (Merchant et al. 2013b; Mendoza et al. 2018; Merchant and de Lafuente 2024), integration, oscillations, and sequence generation (Sussillo and Abbott 2009; Barak et al. 2013; Buzsáki and Wang 2012). Characterizing how different regimes of network activity and neural architectures emerge with different tasks is essential for uncovering general principles of temporal computation in neural systems.

It is unclear whether RNNs converge to a single temporal representation. Similarly, it is unknown whether the encoding of time in biological networks changes with task demands. We expect that the emergence of diverse temporal coding strategies is intimately tied to task complexity, which shapes the neural solution space in non-trivial ways. Increasing task complexity can either constrain the solution space, limiting the diversity of network dynamics that emerge during training (Huang et al. 2024), or result in a rougher optimization landscape with more local minima, potentially promoting a diversity in the dynamics of trained networks (Choromanska et al. 2014; Lyle et al. 2022; Nanda et al. 2023). Which of these effects dominates under realistic training conditions remains unresolved.

Here, we ask how RNNs encode time when trained to perform decision-making tasks with varying demands. We begin with a simple binary decision-making task and progressively introduce additional complexity. This systematic increase in task complexity allows us to isolate how networks represent and use representations of elapsed time across tasks with varying demands. In particular, we ask how task-relevant delay periods (requiring a response after a delay in the absence of an external *cue*) are represented by population activity, how network activity concurrently reflects decision-specific computations, and, ultimately, how internal representations of decision variables are translated into responses. In other words, how does a network represent time while simultaneously preparing and then executing a decision?

Related work has examined the conditions under which RNNs develop sequential versus persistent activity patterns for short-term memory. In particular, Orhan and Ma (2019) systematically varied network and task parameters and showed that fixed versus variable delay structures and architectural choices jointly shape whether memory is implemented via sequences or persistent states. Here we take a complementary angle: rather than asking what form of short-term memory emerges, we ask how elapsed time itself is represented while the network concurrently encodes decision variables. By parameterizing families of delayed decision tasks (fixed vs. variable delays; amplitude- or interval-coded timing; cued vs. uncued integration), we show that RNNs adopt oscillatory or integrative regimes to encode time, with symmetric low-dimensional readout alignment emerging only near decision execution.

We trained multiple recurrent networks (or replicas) on each delayed decision-making task and analyzed the resulting dynamics. Networks converged to oscillatory or non-oscillatory (ramping/decaying) solutions with comparable behavioural performance. Crucially, mirrored responses to positive/negative stimuli were not imposed: with zero-mean initialization and an odd nonlinearity (tanh()), the untrained network is sign-flip equivariant, and our symmetric training set and loss preserve this equivariance. Consistent with this, responses were typically symmetric across stimulus sign, and decision-related activity occupied low-dimensional subspaces. Thus, distinct dynamical regimes can implement the same computation, while population codes remain low-dimensional and (approximately) symmetric across the population.

## 2 Methods

### 2.1 Model

We trained fully connected recurrent neural networks (RNNs) described by Eq. 1 on a sequence of tasks. The dynamics of the RNN model of *N* units is described in terms of the activity column vector **h**. We denote the recurrent weight matrix with **W**^rec^, the input weight matrix with **W**^in^, and the bias term with a column vector **b**. The readout, **z**(*t*), of the network dynamics is given by Eq. 2, where **W**^out^ is the output weight matrix.


(1) $$h\left(t+1\right)=\sigma \left(W^{\text{rec}}h\left(t\right)+b+W^{\text{in}}x\left(t\right)\right),$$

(2) $$z\left(t\right)=W^{\text{out}}h\left(t\right).$$

**Fig. 1:**
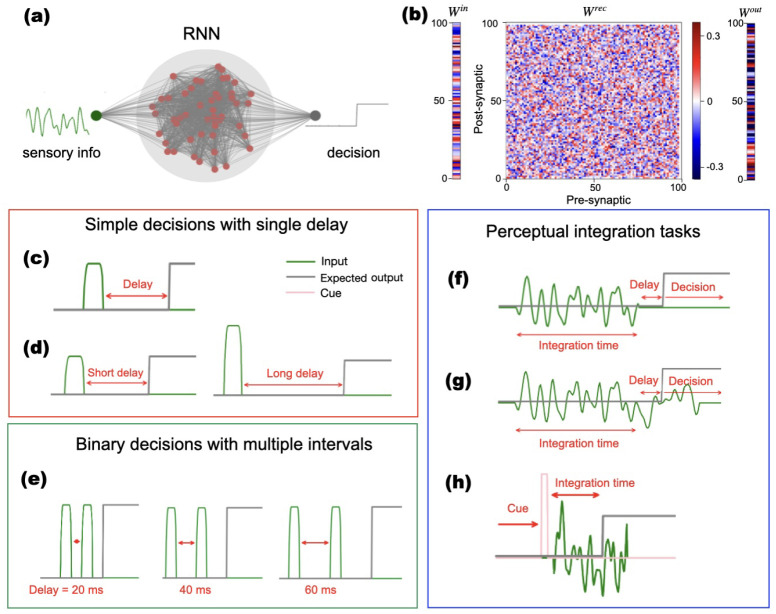
Task parametrization and network architecture. **a)** Schematic representation of the recurrent neural network (RNN) architecture, illustrating both the input and output signals. **b)** Example of post-training RNN with input **W**^in^, recurrent, **W**^rec^, and output, **W**^out^, layers. **c)?h)** Tasks schematics: **c)** Simple Delayed Binary Decisions: Rectangular input pulses sign elicit an output decision. **d)** Context-dependent Binary Decisions: Amplitude-modulated stimuli encoding short/long intervals. **e)** Interval-based Context-dependent Decisions: temporal intervals are encoded via pulse separation. **f)** Windowed Evidence Integration: Signal integration over a predefined window of evidence. **g)** Continuous Evidence Integration: signal integration in a predefined window of a continuous evidence stream **h)** Cued Evidence Integration: Amplitude-based cue defines the duration of evidence integration.

We used a hyperbolic tangent for the activation function, *σ*. Panels a) and b) of Figure 1 illustrate a representation of the network. Each network includes one or more input units, depending on the task, and a single output that presents the decision result based on the computations performed. Our study focused on relatively small networks, containing 100 units, with the weights represented in the connectivity matrices shown in Fig. 1b.

### 2.2 Training

For each of the tasks presented in the bottom panels of Figure 1 and Table 1, we trained a set of 10 RNNs for each initial condition. To ensure our findings were robust across random initializations, we computed each statistic across an ensemble of trained RNNs and monitored its change as new networks were added. We found that all relevant measures varied by less than 5% with the addition of a new sample, indicating convergence. This approach follows a practice similar to that of (Pandarinath et al. 2018), who assessed the consistency of extracted dynamics across multiple model instantiations. During the training process, we allowed all input, recurrent, and output weights to change. Symmetry in the population responses was built in from the initialization (orthogonal or zero-mean random weights) and preserved during training. We employed a supervised learning method based on gradient descent using the adaptive minimization method Adam (Kingma and Ba 2014) with a batch technique. This method and variants have been successfully used to train RNNs on many tasks previously (Yang et al. 2019; Russo et al. 2018; Jarne and Caruso 2023; Jarne and Laje 2023).

For tasks shown in Fig. 1c–e, the stimuli consisted of rectangular pulses with added random noise, amounting to 10% of the pulse amplitude or only random low-amplitude noise (< 10% of stimulus amplitude) when no stimulus was present. Such noise was added to generate trained RNNs that are robust to small fluctuations in input. For integration tasks, shown in Fig. 1f–h, the network was trained to integrate random signals during a time interval and report whether the integrated signal is positive or negative. The integration window can be defined by the time at which the input stops (f), a learned and internally represented duration (g), or cued by an amplitude-based secondary input (h). Within each trial, the stimulus onset time was randomized: The simulation started at time *t* = 0, and the stimulus or cue appeared at a randomly chosen time thereafter up to a maximum time *t* = *t*_*i*_. The target output completed the training set and varied depending on the task. The target was determined by the correct response given the input stimulus, as well as the time at which a response needed to be generated. All tasks could be learned using 100 units and between 20 to 40 epochs over the training dataset, that contained 15,000 time series (trials). Time series length varied between 350 and 500 time points, depending on the task.

Prior to when a response is required by the task [0,T*), we penalized the readout to remain at zero:

$${ℒ}_{\text{pre}}=\lambda_{\text{pre}}\overset{T^{\text{*}}-1}{\underset{t=0}{\sum }}\|z\left(t\right){\|}_{2}^{2},z\left(t\right)=W^{\text{out}}h\left(t\right).$$

For our single-output readout, *z*(*t*) is scalar and this term enforces **z**(*t*) ≈ 0 prior to the response; equivalently, the hidden trajectories evolve roughly in the null space of the readout (orthogonal to **W**^out^) during this interval.

### 2.3 RNN initialization

We used two standard initialization schemes for the recurrent weight matrix, random normal and random orthogonal, to assess the sensitivity of task performance to initial conditions. For each scheme, we trained 10 RNN replicas on the same binary decision-making task and each of the tasks in Table 1. Despite differences in their learned connectivity matrices and eigenvalue spectra, all replicas achieved broadly similar performance levels, suggesting convergence in functional output, if not in internal representations (Jarne and Caruso 2023; Jarne and Laje 2023). All networks were initialized with zero-mean weights (orthogonal or Gaussian) and zero biases, and used an odd nonlinearity (tanh). This combination implies a sign-flip equivariance at initialization?sign-reversed stimuli evoke mirror-reversed population responses. Because the training set and loss were sign-symmetric and we imposed no explicit symmetry constraint, gradient-based training preserved this architectural symmetry. In principle, asymmetry can arise only if the symmetry is explicitly or implicitly broken (e.g., nonzero biases, class imbalance, asymmetric noise/regularization, dropout) or through stochastic/numerical effects; empirically, we observed preservation up to small numerical deviations.

### 2.4 Task parametrization

We considered several versions of delayed binary response tasks with a focus on temporal representation in the networks. The expected responses and the length of the response delay differed depending on the specific task and parameters, as explained in the next sections and summarized in Table 1. The different response delay times corresponded to the elapsed time that had to be learned by the network.

The tasks can be grouped into three categories, which are colour-coded in Table 1. The first category comprises tasks that require a response after a learned delay. The second category comprises tasks that involve a binary decision that needs to be reported after one of multiple learned time intervals. The third category comprises tasks that require the integration of perceptual information over a learned time period, and responses after a learned, but unsignaled time interval.

#### Simple Delayed Binary Decision Making

We first considered a task where the network learns to report the sign of an input signal after a single, learned time interval. This simple delayed report task is based on the two-alternative forced-choice (2AFC) paradigm, which requires selecting between two predefined options in response to a stimulus (Stanislaw and Todorov 1999). We used a single square pulse as input, with the sign of the pulse determining the sign of the correct response. Pulse amplitude was held fixed, but was irrelevant for the task. The network was trained to provide a constant output which matches the sign of the input signal after a fixed, learned delay (see Figure 1c. The delay was learned, and no cues were provided to signal the report time.

#### Context-dependent Binary Decision Making

The context-dependent binary decision-making task reparameterizes the simple delayed binary decision-making task to include two different delay intervals that are signaled by the stimulus amplitude (see Figure 1d). When the signal amplitude is low (high), the delay time is short (long) and equals 50 (100) time units. This design allowed us to ask how the sign of the input signal and elapsed time were represented in the activity in the trained network.

#### Multi-interval Amplitude-based Decision Making

For the multi-interval amplitude-based decision-making task, we used eight distinct stimulus amplitudes, each corresponding to a different time at which a response was to be provided (SI Figure 1). The target output remained binary (positive or negative, depending on the sign of the input signal), but the network had to interpret the stimulus amplitude and keep track of elapsed time to provide a response at the correct, unsignaled time. This design allowed us to ask how the trained network represents the correct response and translates it into a response after a signaled time interval.

#### Multi-interval Distance-based Decision Making

In this task, the required response time was encoded by the time between two consecutive input pulses (See Figure 1e) and (SI Figure 2). Unlike amplitude-based encoding, this approach establishes a more explicit mapping between the input interval and the output timing. To compare these two representations of time, we trained recurrent neural networks on both tasks using the same set of eight distinct intervals. This allowed us to ask how networks represent elapsed time when the length of a time interval is represented in a different way by the input.

#### Time Interval Comparison Task (TICT)

Here, the network compared two temporal intervals (int1 vs. int2) which were presented sequentially and separated by a fixed delay. The target response indicated whether the first or second interval was longer, making this a version of a standard interval discrimination task (Díaz et al. 2025), (see SI Figure 3).

#### Windowed Evidence Integration (Perceptual Decision Making)

Here, the network was required to integrate an input signal over a learned time window. The sign of this integral agreed with the sign of the required response, and the response needed to be reported after a fixed time interval (Figure 1f). No explicit cue was provided to signal when integration needed to cease and a response needed to be generated, but the response needed to be generated at a fixed integral after the cessation of the input.

#### Continuous Evidence Integration

This task was similar to the Windowed Evidence Integration Making task, but the input signal does not end before a response is required (Figure 1g). Rather, the network was trained to respond after a fixed time. Thus, the network had to represent the elapsed time along with the decision variable.

#### Cued Evidence Integration

This was an extension of the Continuous Evidence Integration Decision Making, with an additional cue at trial onset that signaled the duration of the integration time. If there was no cue signal, the network?s output remains zero (Figure 1h). Thus, the network needed to represent elapsed time and translate the decision variable to a response after a signaled time interval that varied from trial to trial.

### 2.5 Analysis

**Fig. 2:**
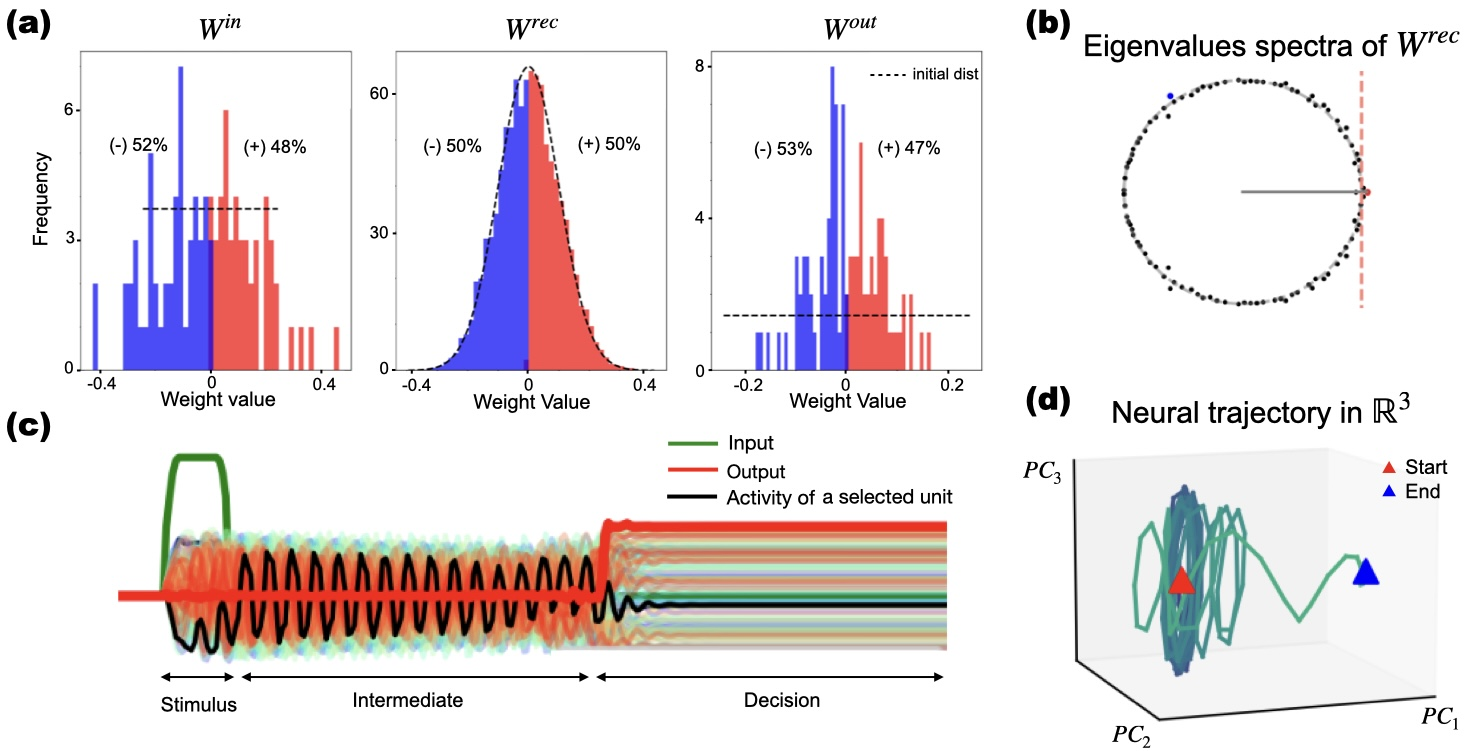
Framework for analyzing trained RNNs. **a)** Histograms with weight distributions: Input **W**^in^, recurrent, **W**^rec^, and output, **W**^out^. Histograms are compared to their respective initialization distributions (e.g., Gaussian or orthogonal). These examples illustrate how training modifies network connectivity. **b)** Spectral analysis of network dynamics: Eigenvalues of the **W**^rec^ matrix are shown in the complex plane. The spectrum relates to the stability and oscillatory activity supported by the network. **c)** Temporal activity across task phases: Example activity traces from a single trial. The green line depicts the stimulus input, the red line shows the network?s output. Colored traces represent the activity of individual units during the stimulus, intermediate, and decision phases. The black line indicates the activity of one highlighted unit. **d)** Low-dimensional representation of neural trajectories: Neural activity of the network during a trial was projected onto the first three principal components. The red triangle marks the start of the trajectory, and the blue triangle marks the end.

Our analyses are summarized in Figure 2. We first examined the structure of the trained network by analysing the connectivity matrices corresponding to the input, recurrent, and output layers (Figure 2a). To understand the network?s dynamical behavior, we analyzed the eigenvalue spectrum of the recurrent weights (Figure 2b), the temporal activity traces during stimulus processing and decision-making (Figure 2c), and visualized low-dimensional projection of the neural trajectories using principal component analysis (Figure 2d). To assess robustness, we ablated internal (recurrent) units by removing the unit from the recurrent graph – i.e., zeroing its incoming and outgoing entries in **W**^Rec^, zeroing its readout weight in **W**_out_, and clamping its state to 0 during simulations -? and then examined the resulting changes in network activity and the spectrum of the modified recurrent connectivity.

To understand the impact of network structure after training on network dynamics and task performance, we examined the eigenvalue distribution of the connectivity matrix (first panel in Figure 2b). This analysis allowed us to relate the solutions obtained across different networks and tasks to the resulting patterns of network activity. Networks with eigenvalues outside the unit circle having substantial imaginary components away from the unit circle tended to support oscillatory or more complex responses, whereas networks with a dominant real eigenvalue far from the unit circle produced gradual, ramp-like or decaying activity (Sussillo and Barak 2013; Jarne 2022). Although RNNs are nonlinear systems, such interpretations are supported by linearizations around fixed points (Sussillo and Barak 2013).

Additionally, we tested the functional importance of individual neurons by performing an ablation analysis on the trained network. Specifically, we systematically disconnected selected neurons by zeroing their incoming and outgoing weights in the recurrent matrix. This allowed us to assess whether task-relevant computation is compartmentalized or distributed, and to identify whether any single unit is critical for network performance.

We also analyzed the patterns of activity, asking whether the population activity is coordinated, random, or correlated, and also how coordinated it is with the output. To capture interactions between units, we computed the correlation matrix of neural activity across time.

We used the generalized correlation measure suggested by Schuessler et al. (2024), which provides a mathematically consistent measure of alignment between the output matrix weights (**W**^out^) and neuronal activity: Consider the neural activity of *N* neurons at *P* time points of the time series stacked into the vector **X**. Then, the corresponding *D*-dimensional output is summarized in the *D* × *P* matrix **Z**, from Eq. 2. The generalized correlation in (Schuessler et al. 2024) is defined as:

(3) $$\rho =\frac{\left\|W_{\text{out}}^{T}X\right\|}{\left\|W_{\text{out}}\right\|\|X\|}$$

where ∥·∥ denotes the Frobenius norm. This quantity measures the global alignment and collective contribution of the neural population to the output. This allows us to classify the computational strategies employed by trained networks. Specifically, this metric distinguishes whether solutions rely on aligned dynamics (where activity patterns directly mirror the output decision axis, implying low-dimensional, interpretable representations) or oblique dynamics (where activity is distributed across higher-dimensional subspaces orthogonal to **W**^out^, reflecting more complex, nonlinear integration).

We computed the generalized correlation between output weights and neural activity across three task periods: the signal or stimulus period, the intermediate delay/signal integration period, and the decision period. Figure 2c shows the activity of selected units (black line) along with the input stimulus (green line) and network output (red line).

We also used principal component analysis (PCA), to generate a low-dimensional representation of the recurrent network’s neural activity, as shown in Figure 2d. The beginning of the temporal trajectory is indicated with a red triangular marker and the end with a blue one. This allows us to compare how the network?s temporal activity trajectories vary in response to different input stimuli.

### 2.6 Non-normality of networks

The connectivity matrix of trained RNNs must deviate sufficiently from a normal matrix to permit transient amplification, a key mechanism for driving the system from its initial state toward task-relevant dynamics (Bondanelli and Ostojic 2020). All trained networks exhibited non-normal recurrent connectivity, as quantified by the Henrici parameter, *d*_*F*_, which captures the degree of deviation from normality,

(4) $$d_{F}\left(W^{\text{Rec}}\right)=\frac{\sqrt{\langle {\left\|W^{\text{Rec}}\right\|}^{2}-\sum_{i=1}^{N}\;{\left|\lambda_{i}\right|}^{2}\rangle }}{\left\|W^{\text{Rec}}\right\|}$$

## 3 Results

Decisions can be the result of a single, temporally localized stimulus, or evidence accumulated over time. Perceptual decision-making typically involves integrating and processing sensory information to guide behavior (Britten et al. 1992; Gold and Shadlen 2007). When decisions are based on accumulated evidence, the process can be separated into two stages. First, incoming sensory input is integrated to form an internal representation, often referred to as a decision variable. If the task imposes a temporal constraint or requires a choice at a certain moment, the decision-making system must also track elapsed time while accumulating evidence. During the integration phase, the decision variable is latent: While it is represented by the network’s internal dynamics, it has little or no impact on behavioural output. For example, a non-human primate may be required to fixate or hold a lever, and only provide a response after a learned time interval (Mendoza et al. 2018). In an artificial neural network with a linear readout, this corresponds to the decision variable being represented by activity in the nullspace of the readout until the decision is made, so that (by design) **z**(*t*) = **W**^out^**h**(*t*) ≈ 0 on [0,T*). When a decision needs to be reported, the neural network must transform the internal representation of the decision variable so that it aligns with the readout, producing an output reflecting the encoded choice.

Responding after a time interval whose length is determined by an input stimulus (Figure 1c,d,e) is different from integrating a sequence of inputs, and providing an answer after a fixed, learned time interval (Figure 1e,f,g). Both tasks require the network to keep track of elapsed time, but it is unclear whether the trained network will represent time in the same way in both cases. The complexity is further increased when these two tasks are combined, and the network needs to integrate an input and provide a response after a time that is determined by the amplitude of an input signal (Figure 1h). While all these tasks required that the networks discriminate between input stimuli, their increasing complexity demanded a potentially different internal dynamics. In the following, we ask how time and decision variables are represented in the activity of the RNN, and how they are translated into a decision after a learned time interval.

### 3.1 Distinct Activity Patterns Result in Equivalent Task Performance

**Fig. 3:**
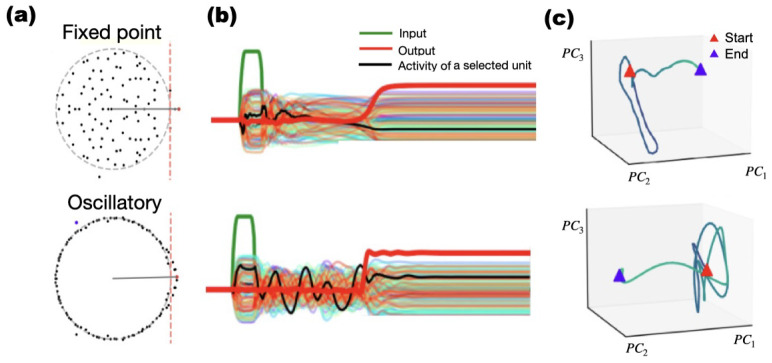
We found that both initializations using an orthogonal and symmetric connectivity matrix can lead to distinct dynamics during the delay. **a)** Eigenvalue spectra of two RNNs showing non-oscillatory (top) and oscillatory (bottom) dynamics. **b)** Input (green), output (red), and unit activity (colored) traces from each network. **c)** Low-dimensional projection of the neural activity (top 3 PCs) showing convergence to the response fixed point after a non-oscillatory transient (Top) and multiple oscillations (Bottom). Red: start, purple: end.

jmM **Distinct dynamical regimes emerge across networks trained on identical tasks.** We observed that trained RNNs used either oscillatory or non-oscillatory (ramping/decaying) transients to represent elapsed time, while achieving equivalent performance on binary decision tasks; after the response, activity settled to fixed points. In Figure 3, the first column a) illustrates the eigenvalue distribution of the connectivity matrix of two networks. The second column b) displays the activity of several individual units, along with the input-output responses for both networks in response to the same stimulus. Finally, the third column c) depicts the activity in neural space, showing the first three principal components. For tasks of the first two categories (Figure 1, panels c, d and e): After training, neurons in the network displayed either oscillatory behaviour, and sometimes an initial activation with slow decay, the first being a little more frequent. For tasks in the **third category** in Table 1, trained networks consistently exhibited oscillatory dynamics. The characteristic frequency and amplitude varied *across replicas* (independent training sessions) and across stimulus conditions, but were stable *across trials* for a given trained network and stimulus.

Networks initialized with random normal and orthogonal weight matrices exhibited distinct spectral signatures (See representative eigenvalue distributions Figure 3a and neural trajectories (PCA space; Figure 3c), demonstrating multiple viable solutions. In the example shown in the figure, the network at the top displays decaying activity, while the one at the bottom exhibits an oscillatory pattern during the delay period. Although pre-training weights do not deterministically dictate the type of dynamics that emerge, orthogonal initializations increased the likelihood of oscillatory solutions.

For simple decision-making tasks, for instance, the Multi-interval Decision Making task, if solutions were oscillatory, elapsed time was represented by the number of oscillation cycles (SI Figure 2a). Trajectories were clustered closely in phase space, with a distance proportional to the value of the time delay (SI Figure 2b).

Similarly, SI Figure 2 shows the distinct activities of two networks trained on the same Multi-Interval-based Context-dependent Decision Making task: One network exhibits ramping dynamics (real dominant eigenvalue), and the second exhibits oscillatory dynamics (complex eigenvalues). The elapsed time since the beginning of the task is represented by the difference in the ramping activity in the first network and the number of oscillation cycles in the second network. This leads to qualitatively different dynamical behaviors as shown by the activity projected onto the three dominant principal components (SI Figure 2c). This representational strategy extended to more complex tasks, such as integration with contextual cues, where cue and non-cue states occupy orthogonal subspaces in PCA space (SI Figure 4). Across tasks, trained RNNs converged either to oscillatory or to non-oscillatory solutions that achieved comparable behavioural performance; we did not test flexible switching between regimes within a single network. Thus, even relatively simple RNNs were trained to solve time-keeping tasks using distinct dynamical behaviours of the type that have been observed in biological neural networks.

### 3.2 Symmetric Stimulus-Responses conserved Across Tasks

**Fig. 4:**
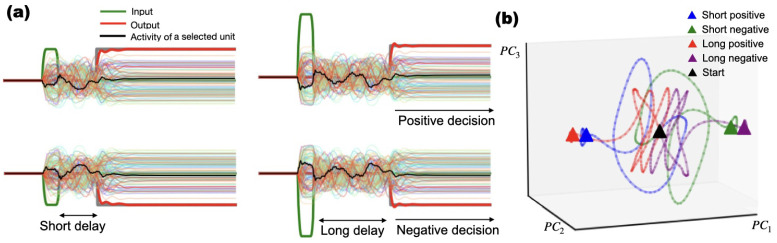
Symmetric internal dynamics in context-dependent binary decision making from Table 1. **a)** Neural responses to stimuli of equal amplitude and opposite sign produce mirror-symmetric unit activity across conditions. The activity of some neurons is shown. The black trace shows an example unit to show that the response inverts sign with stimulus polarity, demonstrating a lack of stimulus-specific specialization. **b)** PCA trajectories for four conditions (“Short/Long” or “Positive/Negative”). Trajectories for opposite-sign stimuli converge to mirrored regions in state space, with consistent clustering across delay intervals.

**Fig. 5:**
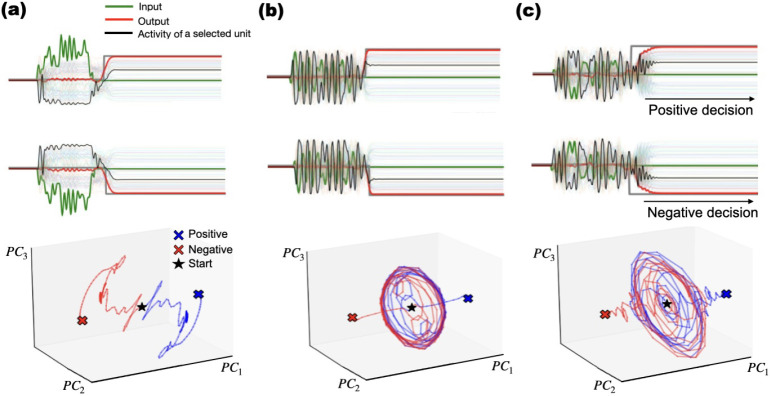
Windowed Evidence Integration. Three different examples (columns **a**, **b** and **c**) of how network activity, including both time series response and projected activity using PCA. The trajectories depend on the characteristics of the signal being integrated. When the random signals are equal and opposite, a symmetrical response is produced. This is also evident in the PCA graph, where the symmetrical trajectories indicate that the behaviour of all units reflects this pattern.

Because zero-mean initialization (orthogonal or Gaussian) with zero biases and an odd nonlinearity (tanh) makes the untrained network sign-flip equivariant, mirrored responses to positive/negative stimuli are expected at initialization. Our contribution is to show that, for symmetric binary tasks, training preserves this architectural symmetry: despite many available asymmetric solutions, optimization typically converged to symmetric ones. We observed that stimuli of equal magnitude but opposite sign evoked mirror-symmetric unit activations. This symmetry was already present in the untrained network dynamics as a direct consequence of our weight initialization scheme. Initializing the recurrent weights with orthogonal matrices or adjusted Gaussian distributions $\left(\sigma \propto 1/\sqrt{N}\right)$ imposes an initial sign-flip equivariance on the network. Combined with the odd symmetry of the tanh activation function, this guarantees that the untrained RNN responds to −*x* with −*h*. Crucially, although never explicitly enforced by the task, this initial symmetry was perfectly preserved throughout training. Trained networks thus continued to respond symmetrically to opposing inputs: units activated by positive stimuli showed inverted patterns of activity for negative stimuli (see Figure 4 and SI Animation 1, 2, and 3). This preserved symmetry generalized across all binary tasks we considered, even for integration (Figure 5). For example, in Context-Dependent Binary Decision Making (Figure 4), units selective for positive stimuli exhibited inverted activity for negative stimuli, and opposite decisions traced mirrored trajectories in principal-component space (Figure 4b), enabling consistent clustering. In the Windowed Integration task, networks also exhibited symmetric responses to sign-flipped inputs, reflecting an inherited exchange symmetry shaped by the task context (SI Figure 4; SI Animation 3). As shown in Figure 5, this symmetry extended across varied inputs. These observations are consistent with population-level “anti-neuron” motifs (Thura et al. 2022), suggesting that functional symmetry can arise via preservation of an initial architectural symmetry during training.

### 3.3 Robust Temporal Processing Across Input Modalities

We next asked whether different input parameterizations?pulse amplitude vs. inter-pulse interval?lead to distinct network dynamics. Comparing the Multi-interval Amplitude- and Distance-based tasks (SI Fig. 1?2), we found that dynamics were *quantitatively similar:* the distributions of the sequentiality index (SI) overlapped across tasks, and the low-dimensional manifolds were closely aligned (small principal angles between the top PC subspaces; see Methods). Any between-task differences were comparable to the trial-to-trial variability observed within a single trained network and stimulus condition.

The tasks being considered differ from one another. The time intervals that need to be “remembered” can be encoded in various ways, such as through the amplitude of the stimulus inputs or the spacing between consecutive stimuli. Decisions can be based on the sign of a stimulus within a defined timeframe or the integration of a signal over a specific window. There may or may not be a cue signal present. Despite these variations, we observe diverse trajectories, none of which are unique to a specific type of task. This suggests that the network’s mechanisms for processing temporal information remain consistent across input forms.

### 3.4 Temporal Decoupling Segregates Sensory Encoding and Decision Execution

**Fig. 6:**
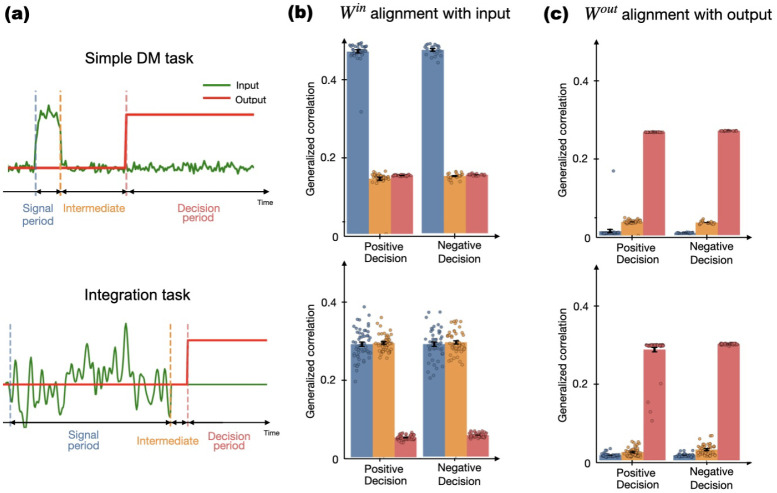
Input-output alignment dynamics (Top simple DM task, bottom integration task). **a)** Task phases (stimulus, intermediate, decision) marked on time series. **b) W**^**in**^ alignment with the input peaks during stimulus phase (0.3?0.5) and decays post-decision. **c) W**^**out**^ alignment with output activity (0.25?0.30) emerges only at decision points.

Input-output alignment (defined by Eq. 3) shifted dynamically across task phases (Figure 6). During stimulus presentation, unit activity was strongly correlated with input weights (**W**^in^; peak correlation ~0.30–0.50), reflecting direct sensory encoding (Figure 6b). This input alignment was present but weaker in integration tasks and dropped to near zero by decision onset. Conversely, output activity aligned with **W**^out^ only at decision execution (correlation ~0.25–0.30; Figure 6c). This decoupling is conserved across tasks (reactive vs. integrative). We speculate that the fact that trajectories only align with the readout near decision time implies a latent, low-dimensional subspace for ”waiting” (during integration or waiting time), which then collapses into the output-aligned manifold – gating sensory evidence until a choice is made.

### 3.5 Distributed Connectivity and Task Performance

**Fig. 7:**
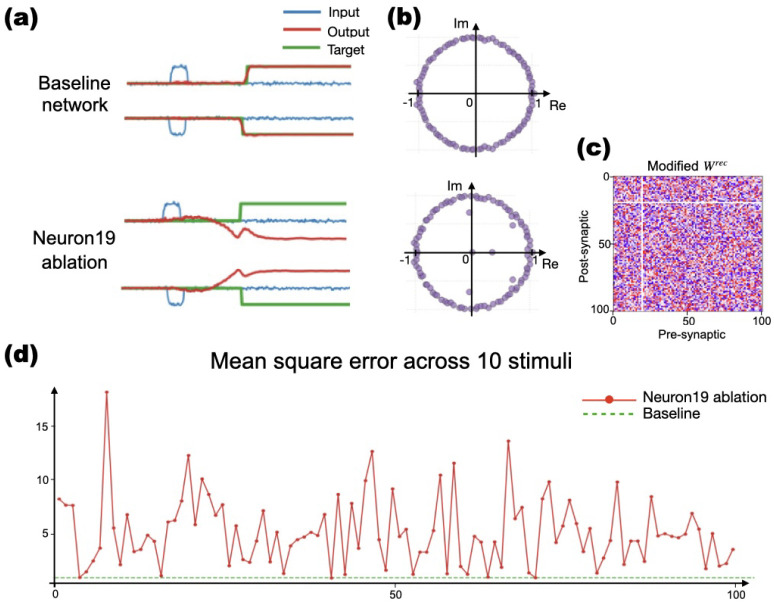
Unit ablation analysis after removing one of the units. **a)** Beseline output activity compared with activity when only one unit is removed. Performance collapses after ablating unit 19, for example. **b)** Changes are also observed in the distribution of eigenvalues. **c)** Trained **W**^rec^ with one unit disconnected. **d)** Mean output-target distance (compared with threshold: 2.5) across 10 stimuli, showing distributed task dependence. Removing any unit, only one of the units at a time, except for a few exceptions from a very small group, collapses the task performance.

Systematic single-unit ablations (one at a time) revealed that network performance relies on distributed coordination rather than modular specialization (See Figure 7). Removing single recurrent units typically produced substantial performance degradation?frequently complete failure?while a small minority of units had milder effects (Fig. 7a,d). Ablations also altered the recurrent eigenvalue distribution (Fig. 7b?c), with similar patterns across replicas and tasks as in Jarne and Caruso (2023). This can be measured by output-target distance, which exceeded 2.5 for over 90% of ablated units (see Figure 7d). This means that most single-unit ablations caused the network?s output to deviate substantially from its intended target across all tested stimuli (see SI animations). These disruptions not only affected responses to certain stimuli but also perturbed global network dynamics, indicating a distributed coding scheme. The 2.5 threshold reflects the maximum target-output distance (meaning distance between the time series of the trained network output and the expected time series output for that stimulus) seen in well-trained networks when averaging over 10 different sample distances of each pair (of fixed length) and comparing the same sample’s target with their output, also in networks with ablation (average of 10 distances also).

Networks tolerated limited ablations of certain units (e.g., unit 15) from a very small group, but performance collapsed when most units (e.g., unit 19), were removed (Figure 7d). Thus, solutions relied on collective dynamics rather than specialized nodes, or subgroups.

### 3.6 Non-Normal Dynamics and Spectral Signatures of Task Complexity

Across tasks, trained networks consistently showed non-zero Henrici parameters (*d*_*F*_ > 0; see SI Figure 5), confirming non-normality and enabling transient amplification; the magnitude of non-normality varied with initialization scheme (random normal vs. orthogonal; SI Figure 6). Task complexity shaped the spectral structure of recurrent dynamics. In integration tasks, eigenvalues were distributed farther from the unit circle (SI Figure 7), supporting a broader range of temporal frequencies and slower decay rates. In the TICT task (SI Figure 3), non-normal dynamics gave rise to oscillatory modes that simultaneously supported time estimation and categorical choice. In both cases, non-normality enabled transient amplification of specific activity patterns?such as timing pulses or evidence ramps?that were selectively boosted before the network settled into a stable state. This mechanism is crucial for encoding and manipulating information during delays and the decision-making processes.

### 3.7 Low-Dimensional Trajectories Encode Decisions and Temporal Context

Projection onto the first three principal components revealed structured, low-dimensional latent trajectories that represented task variables. Across tasks, more than 85% of neural variance was captured by the first three principal components (Figures 3c, 4c, SI Figure 8). Binary decisions were represented by departures from the vicinity of the readout nullspace. Trajectories then approached one of two fixed points symmetric with respect to the readout?s null space; for low-amplitude stimuli, the *first three principal components* explained >90% of the *population-activity variance*. Temporal tasks such as Multi-Interval Decision Making, showed trajectory separation by interval length (SI Figure 1). In cued integration, the presence or absence of a cue shifted trajectories into orthogonal subspaces (SI Figure 4), indicating context-dependent partitioning of network dynamics.

### 3.8 Spectral Signatures Reflect Computational Demands

Eigenvalue spectra reflected computational demands across tasks, consistent with prior work linking spectral geometry to cognitive dynamics (Jarne 2021, 2022; Jarne and Laje 2023). Outlier eigenvalues, which emerged during training, governed network dynamics. Tasks involving integration or timing displayed eigenvalues farther from the unit circle and broader spectral dispersion (SI Figure 7), consistent with the need for richer temporal structure. These spectral patterns were robust to initialization scheme (random vs. orthogonal), with no difference in task performance.

## 4 Discussion

Our findings demonstrate that recurrent neural networks (RNNs), when trained on temporally parameterized decision-making tasks, develop symmetric, low-dimensional dynamics and a variety of functionally equivalent solutions. These findings reflect broader principles of neural computation observed in biological and artificial systems, while also revealing how task complexity and network connectivity shape distinct learning regimes. In particular, we find that eigenvalue dispersion reflects task demands, suggesting a link between dynamical structure and computational load. Below, we situate these findings within recent work on neural dynamics, solution degeneracy, and the rich?lazy spectrum of learning.

The symmetry preserved in unit responses to opposing stimuli–where the same neurons exhibit inverted activity for positive and negative decisions–resonates with findings in Yang et al. (2019), who showed that RNNs trained on multi-task paradigms develop shared, reusable dynamical motifs. Similarly, our observation that decision trajectories cluster in low-dimensional subspaces mirrors the “untangled” population responses observed in motor cortex (Russo et al. 2018). Unlike these studies, here the symmetry was not imposed by architecture or loss: it follows from standard zero-mean initialization with an odd nonlinearity and is preserved by training on a sign-symmetric dataset.

Our findings complement recent work by Driscoll et al. (2024), who demonstrated that recurrent networks flexibly solve multitask demands through shared dynamical motifs and transient trajectories. However, key distinctions emerge in how temporal computations are structured. While their study emphasized cross-task generalization via overlapping neural subspaces, we specifically dissected how task parameterization (e.g., amplitude-modulated delays, cued integration windows) shapes distinct dynamical regimes–oscillatory cycles for timing vs. slowly decaying or integrative dynamics supporting evidence accumulation. Unlike their networks, which reused transient dynamics for rapid task switching, our RNNs developed task-specific spectral signatures (e.g., eigenvalues farther from the unit circle in integration tasks) and symmetric stimulus-response mappings absent in their work. Driscoll et al. (2024) focused on multitask flexibility through shared connectivity motifs, whereas we revealed how degeneracy in single-task solutions arises from structured connectivity (e.g., non-normal matrices enabling transient amplification) and temporal decoupling between input encoding and output alignment.

These observations resonate with Orhan and Ma (2019), who found that fixed delays tend to promote more sequential solutions while variable delays tend to promote persistent activity. In our setting, tasks with fixed response times more often yielded oscillatory “cycle-counting” clocks, whereas tasks that varied the effective delay (e.g., by amplitude or spacing) more often yielded non-oscillatory integrative dynamics. Consistent with the mechanistic picture in Orhan and Ma (2019), oscillatory/sequence-like solutions coincided with more pronounced asymmetric, non-normal recurrent structure (stronger effective ?forward? connections along latent trajectories). Thus, while Orhan and Ma (2019) focused on the form of short-term memory representations, our results highlight how parametrization of delays shapes the *temporal encoding strategies* that RNNs adopt.

The degeneracy of solutions, meaning that distinct networks achieve similar performance through oscillatory or non-oscillatory dynamics, aligns with Huang et al. (2024), who showed that RNNs exploit diverse dynamical motifs to solve identical tasks. Our networks exhibited a spectrum of dynamics with sequentiality indices (SI) values ranging from persistent activity to periodic oscillations, where persistent activity (low SI) and periodic dynamics (high SI) coexist, (see) However, Huang et al. (2024) define complexity through loss landscape roughness, whereas our tasks inherently modulate complexity via temporal integration requirements (e.g., multi-interval vs. cued decisions). We suggest that task structure, not just optimization dynamics, critically influences solution diversity.

While our networks were fully plastic during training, the post-training phase, where recurrent weights (**W**^rec^) remain static, resembled characteristics of “lazy” learning regimes. In such regimes, models remain near initialization with minimal weight updates, as formally defined in Liu et al. (2023), where high-rank initializations promote lazy learning by limiting parameter drift. However, our networks required substantial recurrent weight changes during training to accurately encode temporal intervals, indicating rich learning dynamics in the training phase. Notably, although we used high-rank orthogonal initializations, these did not preclude rich representational development. After training, we observed that readout alignment remained low during integration (*r* = 0) and only sharpened near decision points (*r* = 0.25?0.30), suggesting a hybrid regime: rich learning during training, followed by lazy-like inference using fixed recurrent dynamics and task-specific readout gating. This diverges from the strictly lazy behaviour described in Saxe et al. (2019), and underscores that temporally abstract tasks may necessitate rich internal reconfiguration, even if post-training computation appears static.

The distributed nature of learned solutions?evidenced by substantial performance decrements for most single-unit ablation?echoes principles of degeneracy in biological systems (Edelman and Gally 2001). Unlike specialized circuits in Mante et al. (2013), our networks lacked stimulus-specific units, instead relying on population-wide symmetry. This distributed coding aligns with Stringer et al. (2019)’s observation that high-dimensional neural populations encode stimuli uniformly, but contrasts with modular architectures in Yang and Wang (2020). Notably, our finding that PCA trajectories separate cue-dependent and cue-independent states (SI Figure 4) parallels how prefrontal cortex gates task-relevant information (Russo et al. 2020), suggesting RNNs recapitulate biological gating mechanisms through dynamical (not structural) modularity.

Huang et al. (2024) defines complexity through the loss landscape’s geometry, whereas our work operationalizes it via task demands. For instance, multi-interval tasks required networks to bind temporal information to stimulus identity, increasing computational load compared to binary decisions. This complexity manifested also in eigenvalue spectra: tasks with higher temporal resolution (e.g., amplitude-modulated intervals) produced broader eigenvalue distributions, akin to Jarne (2022). Yet, unlike Yang et al. (2019), where complexity reflects sequential memory depth, our networks optimized for temporal precision through cyclical dynamics, trading off between frequency and phase alignment.

For temporal tasks requiring internal clocks, recurrent weight adjustments (rich dynamics) appear unavoidable, even with high-rank initializations. This complements Braun et al. (2022)’s finding that temporal integration necessitates non-lazy solutions.

## Conclusion

Our results underscore the interplay between task structure, initial connectivity, and learning regimes in shaping RNN dynamics. By integrating insights from rich-lazy theory, solution degeneracy, and biological computation, this work advances our understanding of how artificial and biological networks balance flexibility and efficiency in temporal decision-making. Future work will investigate how these principles-degenerate dynamics, population-wide symmetry, and temporal decoupling-scale to more ethologically relevant tasks, and whether similar signatures exist in biological neural recordings during flexible time-dependent decisions.

## Supplementary Material

Supplement 1

**Supplementary information.** We developed our code in Python based on the TensorFlow (Abadi et al. 2015) and Keras (Chollet et al. 2015) frameworks to train the networks for each task and to perform analysis. The code and trained networks (saved in HDF5 format) can be accessed at https://github.com/katejarne/RNNs_for_DM_and_time_representation

## Figures and Tables

**Table 1:** Summary of all tasks included in the study. Colour coding: Category 1 (red): Simple decisions with single delay. Category 2 (green): Binary decisions with multiple intervals. Category 3 (blue): Perceptual integration tasks.

Task Name	Parameter varied	Figure	Reference	# Intervals	Cue present
Simple Delayed Binary Decision Making	Stimulus sign	Figure 1 c)	(Stanislaw and Todorov 1999)	1	No
Context-dependent Binary Decision Making	Stimulus amplitude	Figure 1 d)	(Mante et al. 2013)	2	No
Multi-interval Amplitude-based Decision Making	Pulse Amplitude	SI Figure 1	N/A	8	No
MultiInterval-based Context-dependent Decisions Making	Pulse distance	1 e)&SI Figure 2	N/A	8	No
Time Interval Comparison Task (TICT)	Comparison between intervals	SI Figure 3	(Díaz et al. 2025)	1	No
Windowed Evidence Integration	None	1 f)	(Newsome et al. 1989; Roitman and Shadlen 2002; Kiani et al. 2008)	1	During fixed window
Continuous Evidence Integration	None	1 g)	(Newsome et al. 1989; Roitman and Shadlen 2002; Kiani et al. 2008)	1	During fixed window
Cued Evidence Integration	Cue and Pulse amplitude	1 h)	(Newsome et al. 1989; Roitman and Shadlen 2002; Kiani et al. 2008)	1	During fixed window
