# Supplementary material for: Task-Parametrized Dynamics: Representation of Time and Decisions in Recurrent Neural Networks
Source: bioRxiv. 2025 Sep 19:2025.09.15.676356. Preprint. [Version 2] doi: 10.1101/2025.09.15.676356 (PMC12458466; doi:10.1101/2025.09.15.676356)
Supplement: Supplement 1 [file media-1.pdf]

Task-Parametrized Dynamics: How Recurrent Networks represent time and decision making through structured connectivity (SI)

Cecilia Jarne[a,b,c], Ryeongkyung Yoon[d], Tahra Eissa[e], Zachary P. Kilpatrick[e], Krešimir Josić[f,g]

[a] *Universidad Nacional de Quilmes, Buenos Aires, Argentina*
[b] *CONICET, Buenos Aires, Argentina*
[c] *Center of Functionally Integrative Neuroscience, Department of Clinical Medicine, Aarhus University, Aarhus, Denmark*
[d] *Department of Mathematics, University of Utah, Salt Lake City, UT, USA*
[e] *Department of Applied Mathematics, University of Colorado Boulder, Boulder, CO, USA*
[f] *Department of Mathematics, University of Houston, Houston, TX, USA*
[g] *Department of Biology and Biochemistry, University of Houston, Houston, TX, USA*

1. Supplementary Figures for tasks from Table 1

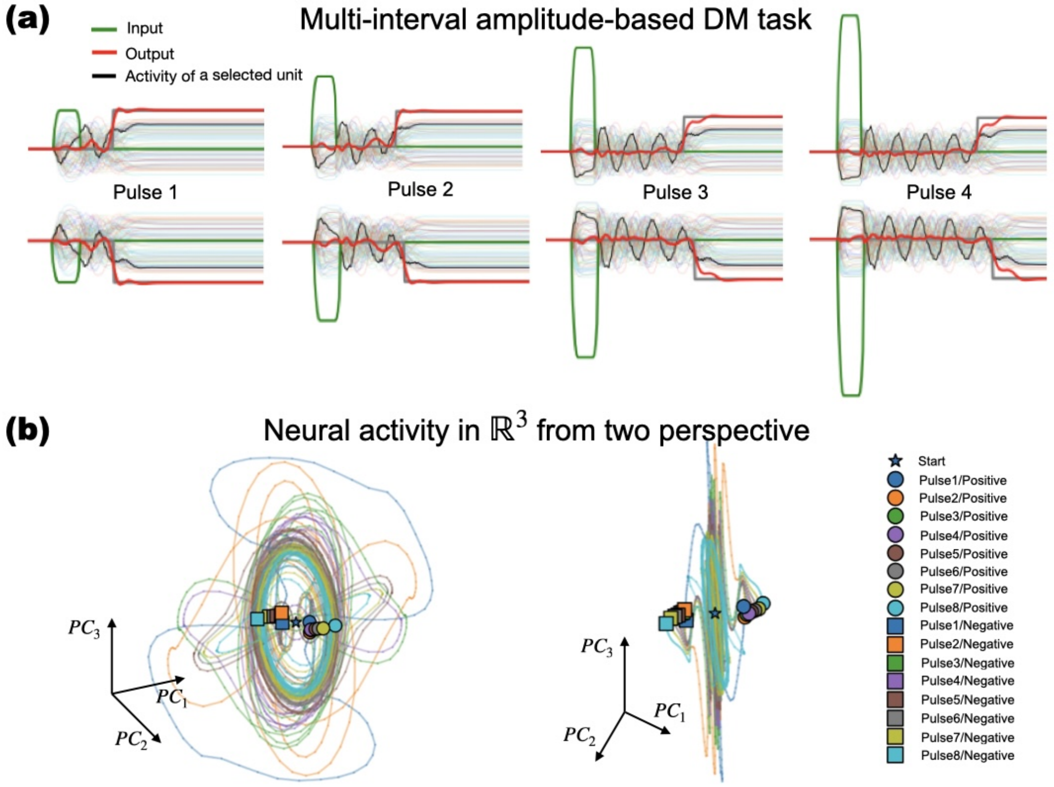

Figure 1: Multi-interval Amplitude-based Decision Making task. It uses eight distinct stimulus amplitudes, each corresponding to a different delay time for target responses. While the output remains binary (positive or negative), the network must store the appropriate response interval based on the stimulus's multiple amplitudes. Panel a) displays four time series with a positive stimulus of varying amplitudes, as well as the network's response to the same stimulus when it is negative. The input signal is represented in green, the output value in red, and the activity of one randomly selected neuron (which is consistent across all series) is highlighted in black. Panel b) illustrates the activity in reduced-dimensionality space (PCA) from two different perspectives, superimposing all possible response time intervals for which the network was trained.

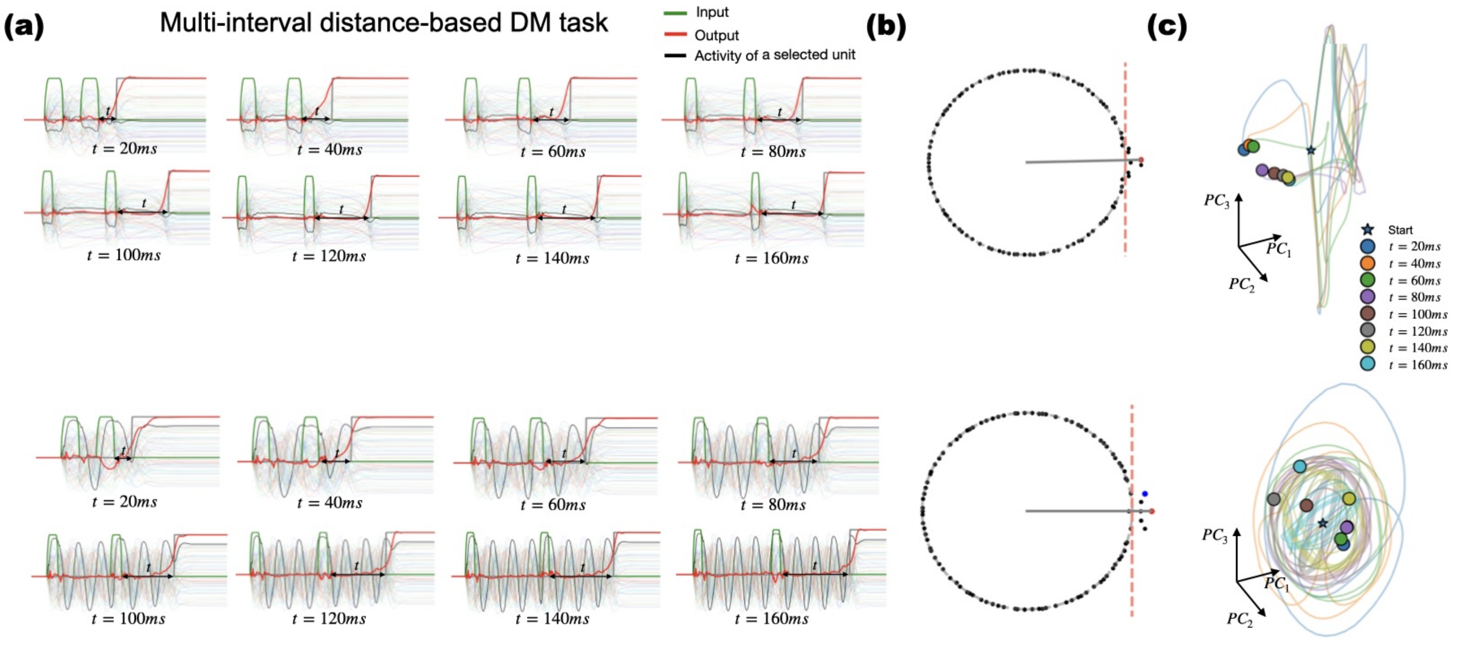

Figure 2: Multi-interval Distance-based Decision Making task. The response time is encoded as the time between two consecutive input pulses. We compared two different networks trained to perform the same task: the Top network and the Bottom network. Column a) presents the eigenvalue decomposition of each trained network. Column b) illustrates the internal behaviour across the eight intervals, highlighting that the Bottom network is primarily characterized by oscillation. Column c) displays the differences in trajectories within the PCA space, showing that different temporal intervals converge at neighbouring points in the PCA representation for each trained network.

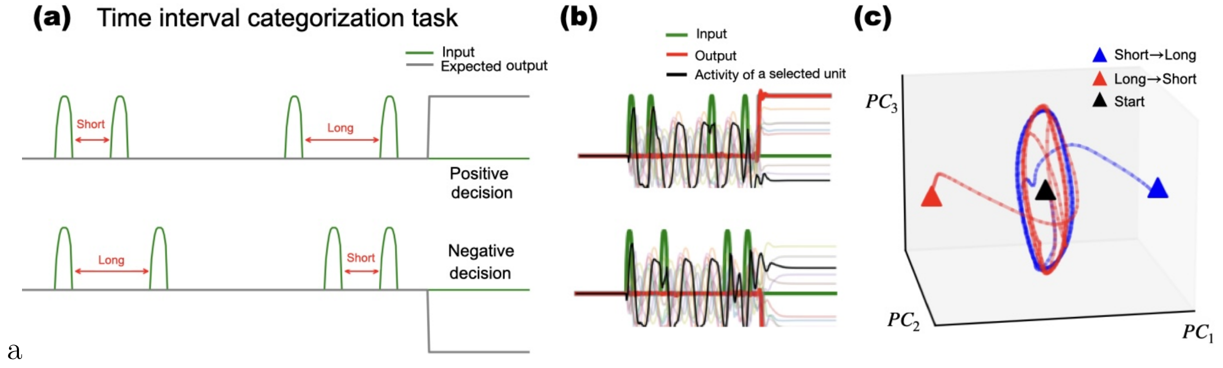

Figure 3: Time-Interval Categorization Task (TICT) dynamics. (a) Task Description. (b) Unit activity and (c) PCA trajectory (bottom). It illustrates oscillatory dynamics encoding time intervals via cycle counts.

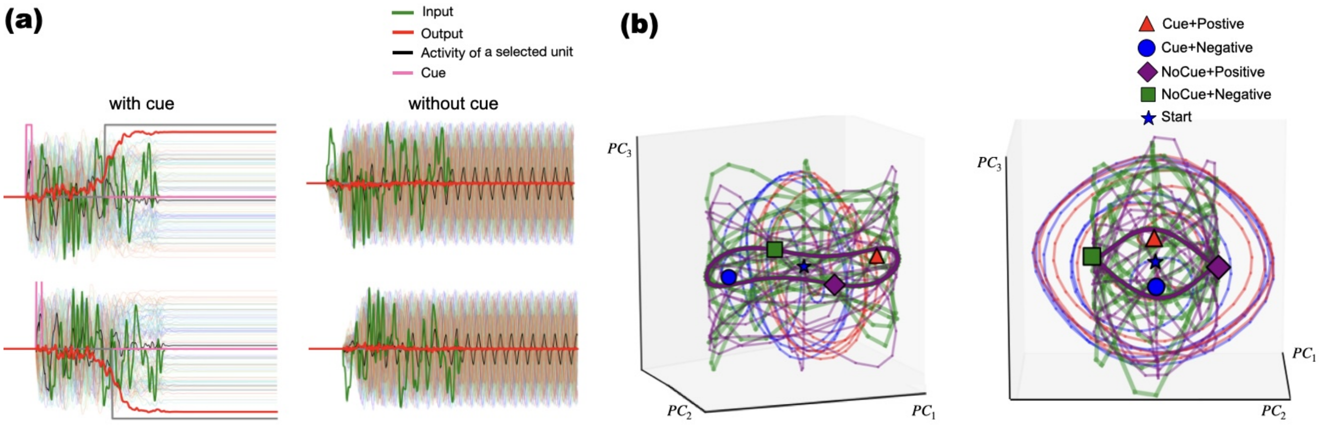

Figure 4: Cued Integration time Decision Making (time series panel a), and PCA panel b): orthogonal PCA planes for cue-present (decision axis) vs. cue-absent (null axis).

2. Supplementary Animations

Files with SI animations are available in:
https://github.com/katejarne/RNNs_for_DM_and_time_representation/tree/main/Animations_compressed

3. Henrici parameter

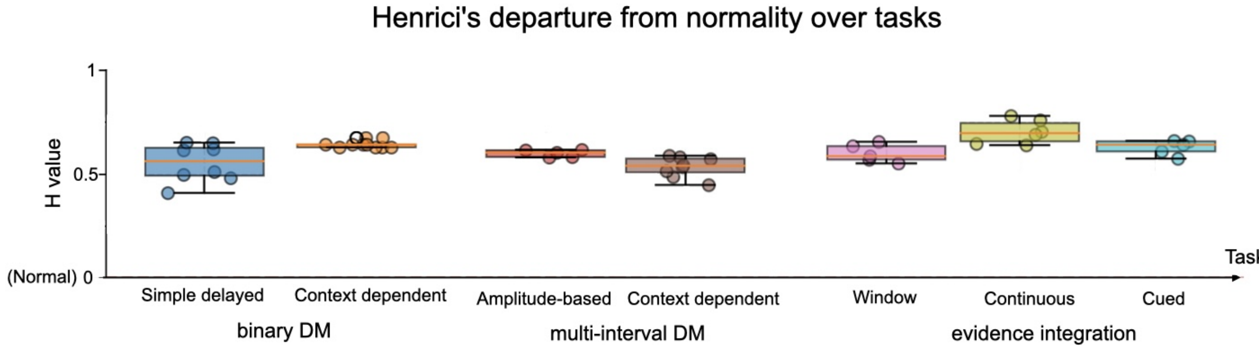

Figure 5: Task-invariant non-normality in trained networks. Henrici parameter (Eq. 4 from main text) quantifies deviation from matrix normality (1: maximal non-normality; 0: normal). Networks across all tasks show consistent non-normal structure with a low H number.

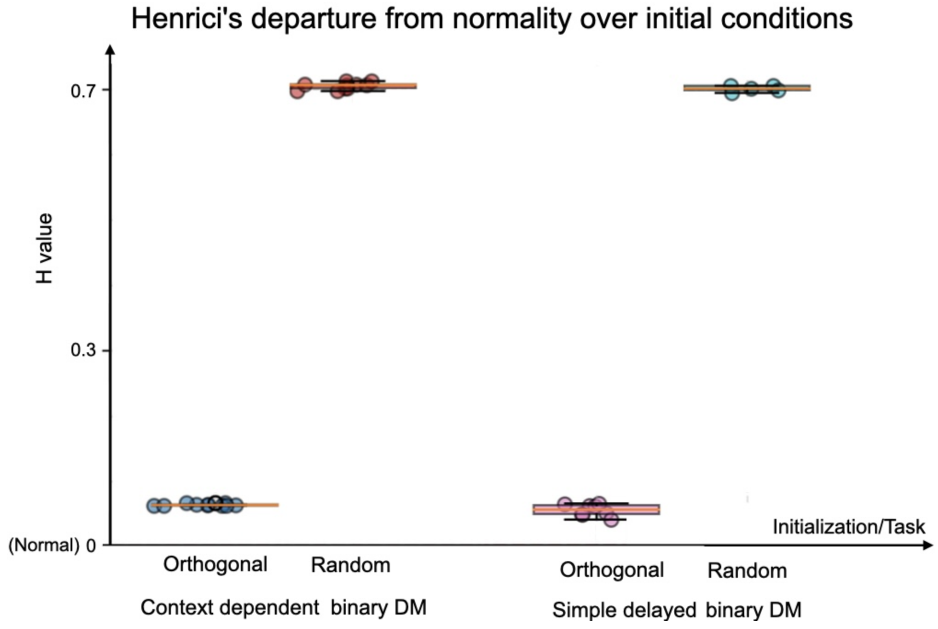

Figure 6: Non-normality in trained networks. We compared both initial conditions before training.

4. Eigenvalue-task relationship

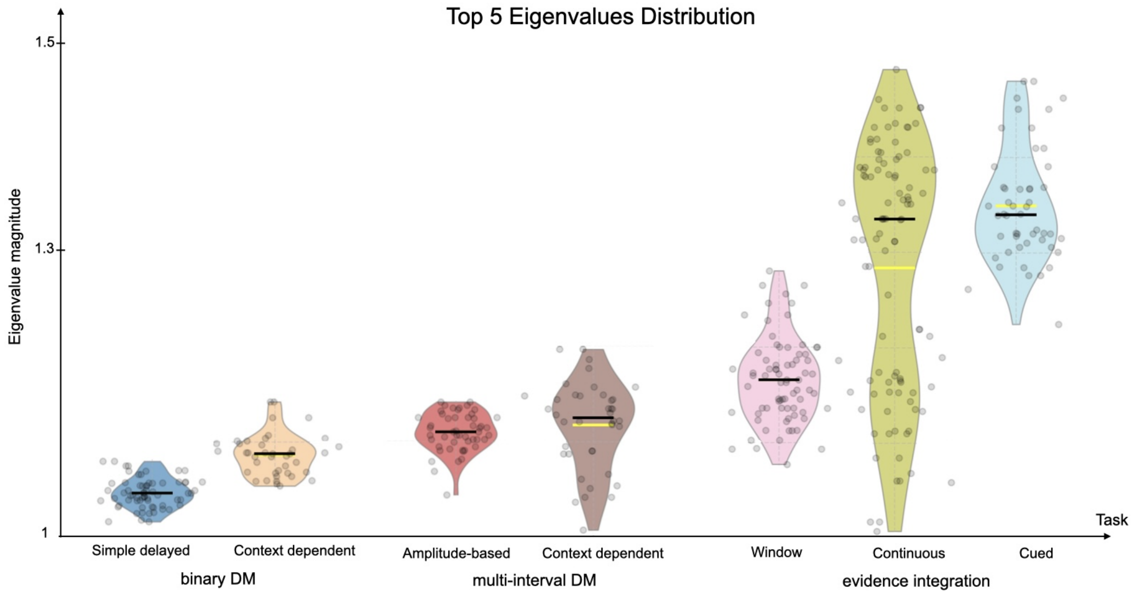

Figure 7: Task complexity dictates eigenvalue positioning. Dominant eigenvalues (5 furthest from unit circle) for networks trained on progressively complex tasks: binary decision (BD), multi-interval (MI), integration (Int), and cued integration (Cued). Integration tasks show eigenvalues at greater distances, reflecting richer frequency interactions.

5. Explained variance

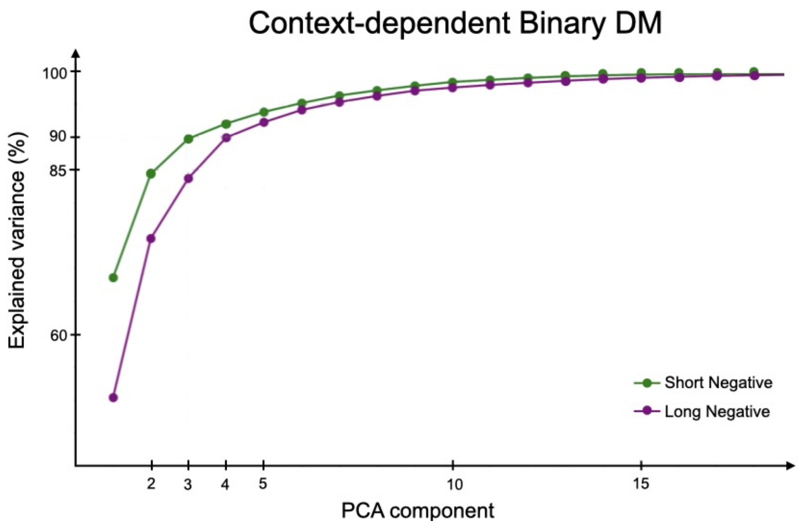

Figure 8: Context-dependent Binary Decision Making. The three principal components explain over 85% variance for high stimuli and 90% for low stimuli.

6. SI index

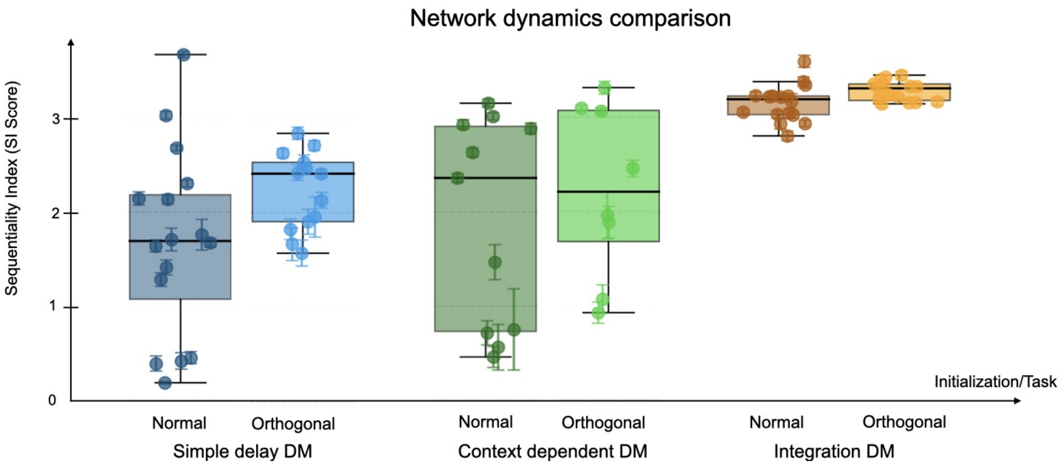

Figure 9: SI index. For the three main tasks considered, and under two initial conditions (random normal and random orthogonal), we estimate the SI value as defined in the work of Huang et al. (2024). This is done by calculating the average of 30 different stimuli for each network. The average reflects the value corresponding to the network, while the error in the average is associated with variations in responses across the different stimuli. Each pair of boxes represents each of the tasks examined. We observe that for the simplest tasks (which do not involve integration), the SI value exhibits greater variation in response classes. In contrast, for tasks that involve integration, a richer frequency behaviour is required to compensate for variations in activity during silent periods. Consequently, the solutions obtained correspond to a superposition of oscillations with different amplitudes and frequencies.
